# Editorial Notes

**Published:** 1948

**Authors:** 


### Editorial Notes

**Medical School Dinner.**

DINNERS have played a large part in local hospital affairs. The first Infirmary Dinner was held at 3 p.m. on December 2nd, 1737, at the "Nagg's Head": this dinner became one of the principal annual events in the city—the accounts were presented, subscriptions solicited and a collection taken. But enthusiasm degenerated into rowdiness and after 1780 the dinners were not held, "because people did not choose to have their heads broke". However, early in the nineteenth century the Faculty held quarterly meetings for dinner and business at the Montague Tavern. From the eighties until after the 1914 war the evening of the inter-hospital Rugger match was devoted to a Staff and Students' dinner: it was abandoned for much the same reasons as operated in 1780.

A proposal is now on foot to hold an Annual Reunion Dinner for ex-students of the Bristol school. If a suitable time can be found it is felt that an annual prize-giving ceremony might take place in the late afternoon: some distinguished alumnus might distribute the prizes won during the preceding year, with the customary speech, explaining that *he* never won a prize or, in fact, attended a lecture. We hope the suggestion will meet with hearty support and that any reader who knows any Bristol men will bring it to their notice.

**The "Journal".**

THE need for economy has led to some changes in our *Journal*. In future we shall not publish the list of Subscribers, of Past Presidents, or of "Books Added to the Library". Examination results will be limited to the names of those completing Second M.B. or receiving degrees or diplomas. In this way we may find space for Local Medical Notes and similar information.

**Almanack.**

SEVERAL times recently two meetings, both of which many practitioners wished to attend, have been held at the same time. To avoid this, as far as possible, Mr. Roberts has kindly agreed to keep an "Almanack" of forthcoming events, so that those who have to arrange meetings in and near Bristol can find out from him what times are likely to be free. The value of such an arrangement depends entirely on the list being complete and up to date, and on notice being given as early as possible of future events. We trust that secretaries of societies, boards and committees will co-operate by sending notice, as long in advance as they can, of all fixtures to the Superintendent, the Medical Library, University of Bristol, and will avail themselves of this service when making their arrangements.

**Books for Hanover.**

GERMAN colleagues have been for long cut off from all knowledge of medical progress and, owing to exchange difficulties, cannot buy English journals and text books. As Bristol has "adopted" Hanover, we are asked to help. Will any readers who can spare recent journals or text books hand them over to the Superintendent of the Medical Library, who will arrange for their despatch to Hanover ?

